# Effects of Phytochemicals on Atherosclerosis: Based on the Gut–Liver Axis

**DOI:** 10.3390/nu18020188

**Published:** 2026-01-06

**Authors:** Yiming Wang, Weiwei Cui

**Affiliations:** Department of Nutrition and Food Hygiene, School of Public Health, Jilin University, Changchun 130021, China

**Keywords:** phytochemicals, atherosclerosis, gut-liver axis, bile acid metabolism, TMAO

## Abstract

**Background**: Atherosclerosis (AS) is the primary pathological basis for cardiovascular and cerebrovascular events, with its development closely linked to dyslipidemia and chronic inflammation. The gut–liver axis, serving as a core bridge connecting gut microbiota, hepatic metabolism, and systemic inflammation, has gained increasing prominence in AS pathogenesis. Phytochemicals exhibit multifaceted biological activities, yet their mechanisms for preventing and treating AS via the gut–liver axis remain to be systematically summarized. This review aims to summarize the potential mechanisms of phytochemicals interventions in AS from an intestinal–hepatic axis perspective. **Methods**: A systematic literature search was conducted using PubMed, Web of Science, and Embase, focusing on previously published articles, reviews, and meta-analyses. Keywords included “phytochemicals”, “flavonoids”, “atherosclerosis”, “AS”, “gut–liver axis”, “gut axis”, “intestinal axis”, “gut microbiota” and “TMAO”. This narrative review synthesizes current research evidence on the interactions among phytochemicals, the gut–liver axis, and atherosclerosis, summarizing their action pathways and molecular mechanisms. **Results**: Phytochemicals (such as polyphenols, carotenoids, saponins, etc.) have low bioavailability but can be metabolized and transformed by gut microbiota. Through multiple mechanisms—including modulating gut microbiota composition, enhancing intestinal barrier function, regulating bile acid metabolism, and exerting anti-inflammatory and antioxidant effects—they positively influence gut–liver axis function. This alleviates lipid metabolism disorders, suppresses systemic inflammation, and thereby combats the onset and progression of atherosclerosis at multiple stages. **Conclusions**: Phytochemicals can intervene in the progression of atherosclerosis through the gut–liver axis. Future studies should further investigate dose–response relationships and conduct clinical validation to determine optimal usage strategies.

## 1. Introduction

The roles of protein, carbohydrates, lipids, and dietary fiber in maintaining health and preventing metabolic diseases have been scientifically confirmed [1]. Beyond traditional nutrients, plant-based foods contain bioactive components known as phytochemicals—including polyphenols, flavonoids, and carotenoids—which are increasingly recognized for their critical role in preventing chronic diseases [2]. Many phytochemicals are difficult to absorb directly in the small intestine, but gut microbiota can convert them into more bioactive metabolites through enzymatic reactions, exhibiting diverse physiological functions such as antioxidant, anti-inflammatory, immunomodulatory, and metabolic regulation effects [3].

Atherosclerosis is a chronic inflammatory disease characterized by plaque formation within the arterial wall, serving as the primary pathological basis for cardiovascular and cerebrovascular diseases [4]. Research indicates cardiovascular disease remains the world’s leading cause of death, claiming 19.2 million lives in 2023. A staggering 79.6% of this disease burden is attributable to modifiable risk factors such as hypertension, poor diet, and high cholesterol [5]. The pathogenesis of atherosclerosis is complex, involving multiple pathways including lipid metabolism disorders, endothelial dysfunction, and immune inflammatory responses [6]. In recent years, growing evidence indicates that the gut microbiota and its interactions with the host via the gut–liver axis play a significant role in the development and progression of atherosclerosis [7].

The gut, the body’s largest digestive organ, is often referred to as the “second brain.” It contains various digestive fluids and serves as a primary organ for nutrient absorption [8]. A complex and dynamic microbial ecosystem inhabits the gut, producing bioactive substances such as short-chain fatty acids, antioxidant enzymes (Catalase, Superoxide dismutase), and bacteriocins (Nisin A, Plantaricin 423). These substances regulate immune responses and metabolic functions, playing a vital role in maintaining intestinal homeostasis and systemic health [9]. The intestinal barrier function is crucial for maintaining this internal environment stability. Composed of epithelial cells, their tight junctions, the mucus layer on the surface, and associated immune components, it precisely regulates the transport of intestinal contents into the body [10]. When the intestinal barrier is compromised and permeability abnormally increases, pathogenic factors such as intestinal bacteria and their byproducts translocate into the bloodstream. This can trigger persistent systemic low-grade inflammation and immune dysfunction, forming a common pathological basis driving various metabolic inflammatory diseases, including atherosclerosis [11].

The liver and intestine are closely interconnected anatomically and functionally, forming a bidirectional “gut–liver axis” via the portal vein and biliary system [12]. As the central hub of lipid metabolism, the liver synthesizes, transforms, and excretes cholesterol while processing and eliminating nutrients, microbial metabolites, and potential toxins from the intestine [13]. Recent studies suggest that gut microbiota and hepatic metabolism may play a role in atherosclerosis pathogenesis. In experimental models, gut microbial metabolites such as short-chain fatty acids have been shown to exert anti-inflammatory effects and may contribute to maintaining vascular endothelial function [14], while trimethylamine *N*-oxide has been shown to impair cholesterol reverse transport and enhance platelet activity, directly accelerating atherosclerosis progression [15,16]. Furthermore, gut microbiota dysbiosis can indirectly influence hepatic and systemic inflammatory responses by regulating bile acid metabolism [17]. Against this backdrop, dietary phytochemicals exhibit substantial potential in preventing and treating atherosclerosis due to their broad anti-inflammatory, antioxidant, and lipid-modulating activities [18]. Notably, many phytochemicals exhibit low bioavailability, and their health benefits largely depend on metabolic conversion by gut microbiota. Cardiovascular protection is achieved indirectly through mechanisms such as modulating microbial composition, improving intestinal barrier function, and intervening in gut–liver axis signaling [19,20].

However, no definitive studies have yet summarized how phytochemicals influence the onset and progression of atherosclerosis via the gut–liver axis. This review systematically examines the interplay between phytochemicals (Table S1), the gut–liver axis, and atherosclerosis. It aims to outline the potential mechanisms by which phytochemicals intervene in atherosclerosis from a gut–liver axis perspective, providing new strategies and theoretical foundations for the prevention and treatment of atherosclerosis.

## 2. Composition and Function of the Gut Microenvironment in Healthy Individuals and Atherosclerosis Patients

**Figure 1:**
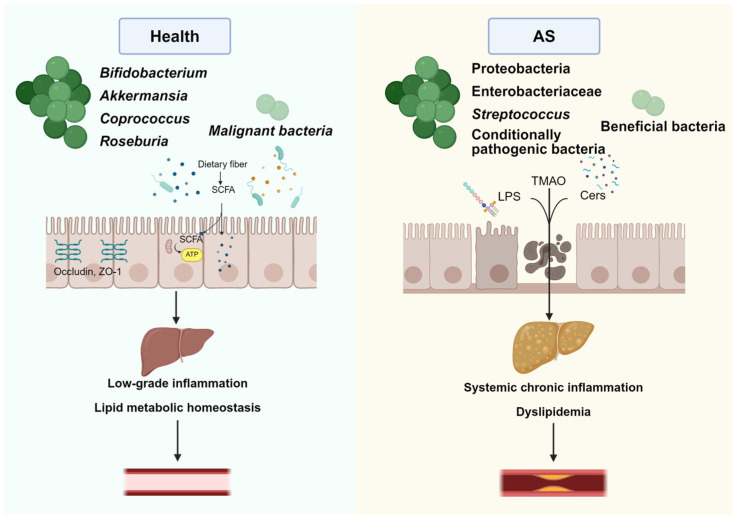
Schematic Diagram Comparing Gut Microbiota Composition, Metabolic Products, and Systemic Effects in Healthy and Atherosclerotic Conditions. Image created by Biorender (https://www.biorender.com).

The gastrointestinal microenvironment plays a crucial role in maintaining health (Figure 1), with its core comprising intestinal epithelial cells, gut microbiota, and the mucosal immune system. Intestinal epithelial cells not only facilitate selective nutrient absorption but also collaborate with the mucosal immune system to form physical and immunological barriers against pathogenic invasion [21]. Furthermore, gut microbiota can sense and respond to host conditions. By dynamically adjusting the secretion of metabolites such as short-chain fatty acids and bile acids, they precisely regulate host metabolic processes and immune homeostasis, thereby serving as key regulators in host physiology [22].

The human gut microbiota is an extraordinarily complex ecosystem comprising bacteria, fungi and viruses, and it plays vital roles in maintaining intestinal barrier integrity, regulating energy metabolism, and sustaining immune homeostasis [23]. The gut harbors an extraordinarily diverse bacterial community, with the phyla *Firmicutes* (primarily comprising Gram-positive *Clostridia*) and *Bacteroidetes* (mainly consisting of Gram-negative bacteria like *Bacteroides fragilis*) accounting for approximately 90% of the gut microbiota [24]. Additionally, the phyla *Actinobacteria*, *Proteobacteria*, and *Verrucomicrobia* also constitute a significant proportion of the gut bacterial community [25].

Compared to healthy individuals, studies reveal that patients with atherosclerosis exhibit lower overall gut microbial diversity and dysbiosis. Specifically, this manifests as increased relative abundance of pro-inflammatory bacterial groups (e.g., *Proteobacteria*, *Streptococcus*) and decreased relative abundance of beneficial bacterial groups (e.g., *Faecalibacterium*, *Bifidobacterium*). However, it should be noted that the current evidence does not consistently show these microbial shifts across all patient cohorts. Factors such as diet, genetics, medication use, and study methodologies contribute to significant heterogeneity in research findings, suggesting that gut microbiota profiles may vary substantially among different populations [26]. Concurrently, the gut microbiota’s metabolic profile undergoes significant alterations, characterized by elevated levels of harmful metabolites and reduced levels of beneficial metabolites: Accumulation of pro-inflammatory metabolites like trimethylamine *N*-oxide (TMAO) and imidazolepropionic acid (ImP) exacerbates vascular inflammatory responses; conversely, levels of anti-inflammatory short-chain fatty acids (SCFAs) show a marked decrease [27,28,29]. Metabolic disruption of serum deoxycholic acid (DCA) has also been observed, a process potentially promoting thrombosis by affecting platelet function [30]. Furthermore, atherosclerotic patients may experience intestinal permeability, allowing the harmful substance lipopolysaccharide (LPS) to enter the bloodstream and trigger systemic chronic inflammation, thereby accelerating disease progression [26].

Gut microbiota perform multifaceted roles, regulating the intestinal microenvironment and influencing human health. Regarding nutrient metabolism, humans lack enzymes to digest most dietary fiber. Bacteria such as *Bacteroidetes* and *Firmicutes* in the gut assist in breaking down dietary fiber, producing short-chain fatty acids like acetate, propionate, and butyrate [31]. Butyrate serves as the primary energy source for colonic epithelial cells, playing a crucial role in maintaining intestinal health [32]. Propionic acid produced by gut microbes can enter the liver to inhibit cholesterol synthesis and gluconeogenesis, thereby aiding in blood glucose and lipid regulation [33]. Gut microbes interact with the intestinal barrier: on one hand, they synthesize butyrate to energize colonic epithelial cells, ensuring their proliferation and repair while inducing the formation of epithelial defense barriers to maintain intestinal barrier integrity [34]. Conversely, an intact intestinal barrier controls bacterial diversity and distribution by releasing antimicrobial peptides (AMPs) and secretory immunoglobulin A (sIgA), directly killing bacteria or regulating their behavior to maintain gut microbial equilibrium [35,36]. Furthermore, gut microbiota can directly promote the onset and progression of vascular dysfunction and atherosclerosis by influencing inflammatory responses, lipid metabolism, and blood pressure regulation [37].

## 3. The Role of the Gut–Liver Axis in Atherosclerosis

**Figure 2:**
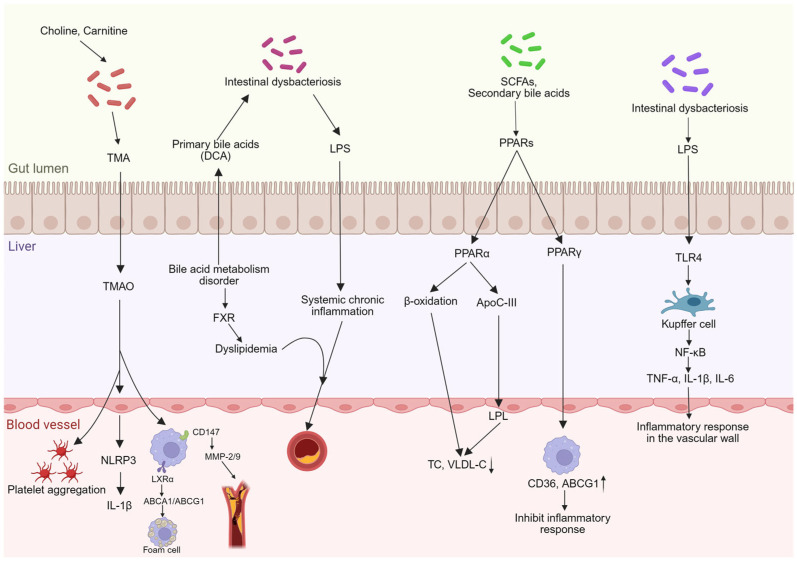
The Role of the Gut–Liver Axis in Atherosclerosis. The arrows indicate a decrease (↓) or increase (↑) in the respective parameter. Image created by Biorender (https://www.biorender.com).

The gut–liver axis can influence atherosclerosis by affecting gut microbiota balance, intestinal barrier function, bile acid metabolism, lipid metabolism, and inflammatory responses (Figure 2).

### 3.1. Gut Microbiota Dysbiosis and Barrier Impairment

Gut microbiota metabolize dietary fiber to produce SCFAs, with acetate entering the liver to fuel lipid and cholesterol synthesis [38]. Propionate reduces cholesterol synthesis by inhibiting 3-hydroxy-3-methylglutaryl-coenzyme A (HMG-CoA) reductase activity and binds to specific receptors on intestinal epithelial cells to exert systemic metabolic regulation [39]. Butyrate promotes cholesterol reverse transport from peripheral cells to the liver and acts as a histone deacetylase (HDAC) inhibitor, playing a key role in immune regulation and suppressing vascular inflammation [40]. Thus, the gut microbiota can modulate cholesterol synthesis, lipid metabolism, and inflammatory responses by influencing SCFA levels, thereby inhibiting atherosclerosis progression.

The gut microbiota also converts choline and carnitine derivatives into trimethylamine (TMA), which enters the liver and is oxidized into TMAO [41]. TMAO directly activates NLRP3 inflammasomes within vascular endothelial cells and immune cells, triggering massive release of proinflammatory factors like interleukin-1β (IL-1β) to initiate and sustain vascular inflammation [42]. In macrophages, TMAO interferes with the function of liver X receptor alpha (LXRα) and downregulates the expression of its downstream ATP-binding cassette transporter A1/G1 (ABCA1/ABCG1), transforming cells into foam cells and promoting atherosclerosis progression [43]. Furthermore, a research team from Southern Medical University discovered that TMAO stimulates macrophages to increase expression of the surface protein CD147. Its upregulation further leads to significantly elevated expression and activity of matrix metalloproteinase-2 (MMP-2) and matrix metalloproteinase-9 (MMP-9). Its degradation causes the fibrous cap to thin and become fragile, making the plaque more prone to rupture [44]. TMAO further stimulates platelets, causing more intense release of intracellular calcium ions upon stimulation and thereby enhancing platelet aggregation activity. This induces a hypercoagulable state in the blood, making thrombus formation highly likely at the site of atherosclerotic plaque rupture, triggering acute myocardial infarction or stroke [45].

Concurrently, gut bacteria metabolize phenylalanine from dietary sources into phenylacetylglutamine, which affects platelet activity and coagulation function, accelerating the progression of atherosclerotic [46]. Dysbiosis of the gut microbiota increases harmful substances within the intestine, compromising the intestinal barrier function and immune system, leading to metabolic disorders and hastening the development of atherosclerotic.

### 3.2. Regulation of Bile Acid Metabolism

Bile acid metabolism is a crucial component of the gut–liver axis, and research demonstrates its close association with atherosclerotic progression. In healthy individuals, excess cholesterol is degraded daily through the enterohepatic circulation to inhibit atherosclerotic formation, whereas atherosclerotic patients exhibit disrupted bile acid metabolism [47]. Bile acids themselves serve as crucial signaling molecules, activating nuclear receptors like FXR throughout the liver and intestines to broadly regulate host glucose, lipid metabolism, and energy balance [48]. Abnormal bile acid metabolism is associated with increased intestinal permeability, which may facilitate LPS entry into the bloodstream and contribute to systemic chronic low-grade inflammation. Additionally, certain secondary bile acids (DCA, LCA) have been implicated in pro-inflammatory responses, and their elevation often coincides with reduced abundance of beneficial bacteria such as *Akkermansia*. These interconnected disturbances in the gut microenvironment are believed to collectively promote the development and progression of atherosclerosis [49]. Bile acids also participate in regulating gut microbiota composition and influence atherosclerotic development via the gut–liver axis. Upon entering the intestine, the fate of primary bile acids is primarily governed by the microbiota, particularly through their bile salt hydrolase (BSH) enzymes, which convert primary bile acids into secondary bile acids [50]. Simultaneously, hydrophobic bile acids like DCA accumulate within bacterial cells, disrupting cell membranes and damaging DNA to inhibit bacterial growth, thereby directly shaping the microbial community structure [51]. Dysbiosis may reduce anti-inflammatory or metabolically beneficial bile acids while increasing pro-inflammatory ones. This altered bile acid profile, through FXR and TGR5 receptor mechanisms, may exacerbate lipid metabolism disorders and chronic inflammation, thereby promoting the onset and progression of atherosclerotic [52,53,54].

### 3.3. Lipid Metabolism

Dysregulated lipid metabolism is a significant risk factor for AS. Lipid metabolism encompasses three pathways: exogenous metabolism, endogenous metabolism, and reverse cholesterol transport (RCT). Peroxisome proliferator-activated receptors (PPARs), as ligand-activated receptors within the nuclear hormone receptor family, are highly expressed in tissues such as the liver, intestine, kidney, and vascular walls. The PPAR family comprises three subtypes: PPARα, PPARδ, and PPARγ. Short-chain fatty acids produced by gut microbiota fermentation of dietary fiber, along with secondary bile acids, can directly or indirectly activate hepatic PPARα. This promotes hepatic uptake and β-oxidation of fatty acids, thereby reducing the fatty acid substrate pool available for triglyceride synthesis. Simultaneously, it reduces Apolipoprotein C3 (ApoC-III) expression and enhances lipoprotein lipase activity, significantly lowering plasma triglyceride and Very Low Density Lipoprotein (VLDL) levels to decrease the number of atherogenic lipoproteins at the source [55,56]. Butyrate also exerts protective effects in atherosclerosis models by activating PPARγ to upregulate Cluster of differentiation 36 (CD36) and ABCG1 expression, promoting cholesterol efflux in macrophages and suppressing inflammatory responses [57]. Concurrently, PPARγ activation enhances adipocyte differentiation, increases fatty acid uptake and storage in adipose tissue, and improves systemic insulin sensitivity [58].

### 3.4. Inflammatory Response

Intravascular inflammation within the arterial wall characterizes atherosclerosis, involving macrophage accumulation and activation [59]. Dysbiosis disrupts intestinal barrier integrity, leading to translocation of bacterial metabolites from the gut, triggering enteritis, and potentially impairing normal liver function [60]. When the intestinal barrier is compromised, bacterial products like LPS translocate to the liver via the portal vein. LPS binds to the TLR4 receptor, activating Kupffer cells in the liver. This initiates intracellular signaling pathways, including NF-κB, leading to the release of pro-inflammatory cytokines such as tumor necrosis factor-α (TNF-α), interleukin-1β (IL-1β), and interleukin-6 (IL-6), thereby inducing hepatic inflammation [61]. Simultaneously, these pro-inflammatory factors produced by the liver enter the systemic circulation, promoting vascular wall inflammation and accelerating the formation and progression of atherosclerotic plaques [62].

## 4. Mechanisms of Phytochemicals Affecting the Gut–Liver Axis

Phytochemicals are naturally occurring bioactive substances in plants. While not essential nutrients for human survival, they play a crucial role in maintaining health and preventing disease. Common phytochemicals include polyphenols, carotenoids, terpenoids, organosulfur compounds, saponins, phytoestrogens, phytate, and phytosterols, exhibiting diverse biological effects such as antioxidant, antibacterial, anticancer, antiallergic, and anti-inflammatory properties. For instance, diterpenoids extracted from white horehound (*Glycyrrhiza glabra*) exert anti-inflammatory effects by inhibiting nitric oxide (NO) production, thereby indirectly influencing systemic inflammatory responses [63]. Gut microbiota can metabolize phytochemicals into active small molecules such as 3,4-dihydroxybenzoic acid [64], short-chain fatty acids [65], and various phenolic acids. Notably, polyphenolic phytochemicals can be fermented by gut microbiota to produce short-chain fatty acids, which are key substances for maintaining intestinal barrier integrity and suppressing intestinal inflammation [66]. These substances exert significant physiological regulatory roles in the human body, influencing the gut–liver axis through mechanisms such as modulating gut microbiota, repairing the intestinal barrier, and affecting related signaling pathways and metabolic products.

### 4.1. Modulating Gut Microbiota Structure

Phytochemicals influence overall metabolic output by modulating the structure and composition of the gut microbial community. These metabolic changes exert profound effects on gut–liver axis function through a series of complex signaling pathways. Green tea polyphenols, particularly epigallocatechin gallate (EGCG), have been shown to protect gut health by significantly increasing the abundance of the beneficial bacterium *Akkermansia muciniphila*. They also inhibit harmful bacteria from producing endotoxins, thereby reducing endotoxemia and improving chronic low-grade inflammation, which indirectly affects the liver [67]. EGCG also inhibits lipid synthesis, alleviates hepatic steatosis, and reduces oxidative stress by activating the liver’s AMPK signaling pathway [68]. Licorice flavonoids (LFs), particularly bioactive compounds like Licochalcone A, Licochalcone B and Liquiritigenin, can partially reverse ethanol-induced dysbiosis by promoting the growth of beneficial microbiota and increasing levels of short-chain fatty acids (SCFAs) such as butyrate, propionate, and valerate in the gut. Concurrently, the study revealed that certain microbial genera significantly increased in gastric ulcer model groups showed negative correlations with these SCFA levels, suggesting licorice flavonoids may further optimize microbial composition by suppressing these harmful bacteria [69]. SCFAs produced by gut microbiota play a crucial role in maintaining liver health. Butyrate activates the hepatic LKB1/AMPK signaling pathway and enhances expression of the endoplasmic reticulum-anchored protein Insig, thereby inhibiting the cleavage activation of SREBP-1c and downregulating the expression of lipid synthesis-related genes, ultimately reducing fat accumulation in the liver at its source [70]. Furthermore, resveratrol reduces liver fibrosis markers (hydroxyproline content, collagen deposition) and improves liver function indicators by inhibiting non-pathogenic staphylococcal growth, suppressing bacterial translocation, and mitigating intestinal barrier damage [71].

### 4.2. Repairing and Enhancing the Intestinal Barrier

The intestinal barrier functions with significant biological importance in the gut–liver axis, precisely regulating material and information exchange between the gut and liver. A systematic review published in *Nutrients* indicates that anthocyanins reduce intestinal permeability by promoting SCFAs acid production from gut microbiota to energize intestinal epithelial cells, stimulating mucus secretion and antimicrobial peptide expression, increasing tight junction proteins and goblet cell numbers, and activating the NLRP6 pathway to maintain immune homeostasis. This reduces intestinal leakage and alleviates hepatic exposure to harmful substances [72]. Concurrently, berberine improves epithelial and mucosal barrier function, reduces intestinal inflammation and oxidative stress [73], and decreases endotoxin entry into the bloodstream, ultimately alleviating liver inflammation and damage [74]. Resveratrol upregulates key tight junction proteins such as occludin, ZO-1, and claudin-1, thereby decreasing intestinal permeability and reducing blood endotoxin levels. It directly inhibits the activation of downstream inflammatory pathways in the liver, including FAK/MyD88/IRAK4, thereby alleviating hepatic inflammatory responses [75].

### 4.3. Regulation of Bile Acid Metabolism

Certain phytochemicals precisely regulate bile acid metabolism through multi-targeted, cross-organ synergistic actions. In the liver, ginsenosides (Rg1, GF2) downregulate the expression of Cholesterol 7-alpha hydroxylase (CYP7A1), the rate-limiting enzyme in bile acid synthesis, by modulating nuclear receptors such as the FXR. Simultaneously, they upregulate the expression of transporters like the bile acid efflux pump BSEP and MRP2, thereby inhibiting excessive bile acid synthesis and promoting its excretion to maintain hepatic bile acid homeostasis [76]. In the gut, ginsenosides (Rh4, Rg1) enhance the activity of gut bile acid hydrolases and 7α-hydroxysteroid dehydrogenase by enriching the mucus-loving bacterium *Akkermansia*, promoting the conversion of primary bile acids to secondary bile acids, and increasing the proportion of hepatoprotective bile acids like ursodeoxycholic acid [77]. Furthermore, berberine enhances the liver’s cholesterol-to-bile acid conversion capacity by promoting *Akkermansia* growth and suppressing AMPK-mediated upregulation of CYP7A1 expression [78]. Quercetin, widely present in fruits, promotes *Akkermansia* colonization. Its metabolite indole-3-lactic acid (ILA) upregulates CYP8B1 expression via the FTO/m6A/YTHDF2 signaling pathway, increasing cholic acid production and subsequently activating FXR in adipose tissue to inhibit lipid accumulation [79].

### 4.4. Modulating Anti-Inflammatory and Antioxidant Responses

Ginsenosides influence the gut–liver axis not only by modulating gut microbiota composition, repairing the intestinal barrier, and regulating bile acids, but also by inhibiting the LPS/TLR4/NF-κB inflammatory pathway [80], activating the Sirt1/Nrf2 antioxidant pathway [81], thereby alleviating inflammation and oxidative damage caused by abnormal bile acid accumulation. These findings suggest that Rh4 may improve hepatic metabolic disorders, such as cholestasis and non-alcoholic fatty liver disease, through mechanisms involving gut microbiota and bile acid metabolism [77]. Research findings indicate that sulforaphane alleviates systemic inflammation by reducing LPS levels, subsequently inhibiting the phosphorylation of c-Jun *N*-terminal kinase (JNK). JNK inactivation promotes phosphorylation of insulin receptor substrate (IRS) tyrosine sites, thereby activating protein kinase B (AKT) and ultimately initiating insulin signaling. This pathway activation accelerates hepatic glycogenesis by promoting phosphorylation of glycogen synthase kinase 3β (GSK3β) while simultaneously inhibiting the activity of glucose-6-phosphatase (G6PC), phosphoenolpyruvate carboxykinase 1 (PCK1), and forkhead box protein O1 (FOXO1), thereby effectively suppressing hepatic gluconeogenesis [82].

## 5. Phytochemicals Influence Atherosclerosis Through the Gut–Liver Axis

**Figure 3:**
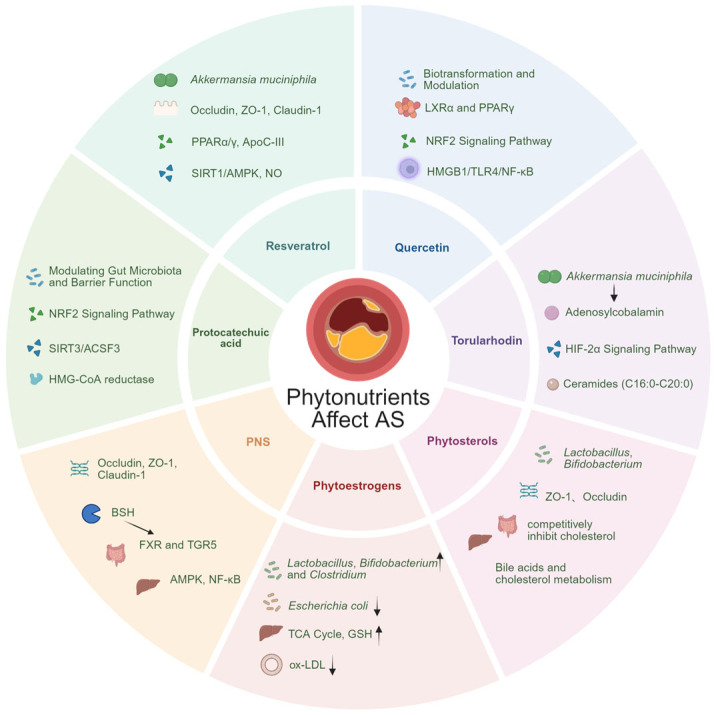
Phytochemicals Influence Atherosclerosis Through the Gut–Liver Axis. The arrows indicate a decrease (↓) or increase (↑) in the respective parameter. Image created by Biorender (https://www.biorender.com).

Phytochemicals intervene in the progression of atherosclerosis through multiple pathways via the gut–liver axis. Their mechanisms of action primarily include regulating gut microbiota composition, enhancing intestinal barrier function, modulating bile acid metabolism, and exerting anti-inflammatory and antioxidant effects (Figure 3). For detailed mechanisms, see Table S4 in Supplementary Materials.

Despite structural differences among various phytochemicals, they share significant common mechanisms for intervening in atherosclerosis via the gut–liver axis. These primarily include reshaping the gut microbiota, enhancing intestinal barrier function, regulating microbial metabolites, and exerting systemic anti-inflammatory and antioxidant effects. Despite these commonalities, distinct characteristics emerge among compound classes: polyphenols (e.g., resveratrol) exhibit health effects highly dependent on microbial metabolism with marked interindividual variability; saponins (e.g., ginsenosides) demonstrate particular efficacy in regulating bile acid metabolism and nuclear receptors; while phytosterols primarily inhibit cholesterol absorption through structural competition. In summary, these compounds collectively follow an integrated framework: they are first metabolized by gut microbiota to optimize microbial composition, thereby repairing the intestinal barrier and reducing endotoxin translocation; the resulting signals and metabolites influence the liver via the portal vein, regulating lipid metabolism and inflammatory pathways; ultimately, they systemically improve blood lipids and reduce vascular inflammation, thereby inhibiting AS progression.

### 5.1. Polyphenolic Compounds

Protocatechuic acid (PCA), also known as 3,4-dihydroxybenzoic acid, is widely found in plants such as the black fern and holly. Notably, it is also an important polyphenolic compound with a well-defined content in pomegranate peel. PCA helps maintain a healthy gut microbiota structure, which in turn reduces the production of harmful metabolic byproducts. In a study of LPS-challenged weaned piglets, dietary supplementation with 4000 mg/kg (0.4%) PCA effectively up-regulated the expression of intestinal tight junction proteins (such as ZO-1 and claudin-1), enhancing intestinal barrier integrity. This reduced LPS entry into the bloodstream and significantly decreased serum levels of inflammatory mediators including IL-6 and TNF-α, effectively preventing systemic inflammation [83]. Concurrently, PCA alleviates ferroptosis, hepatic lipotoxicity, and steatosis by activating the NRF2 signaling pathway, potentially improving hepatic metabolic function, reducing oxidative stress levels, and decreasing lipid peroxidation [84]. It can also alleviate high-fat diet-induced hepatic lipid accumulation by regulating the SIRT3/ACSF3 pathway, thereby addressing lipid metabolism disorders at their root cause [85]. PCA inhibits cholesterol absorption in the gut while promoting bile acid excretion. Within the liver, it suppresses the activity of HMG-CoA reductase, a key enzyme in cholesterol synthesis, thereby reducing cholesterol production at its source [86]. In summary, PCA delivers comprehensive benefits through multiple pathways—including fat reduction, cholesterol lowering, anti-inflammatory effects, and antioxidant activity—thereby reducing risk factors for atherosclerosis at their source and protecting the cardiovascular system.

Resveratrol can function as a prebiotic, selectively promoting the growth of certain beneficial bacteria. Multiple studies indicate it increases the abundance of beneficial bacteria such as *Akkermansia*. Simultaneously, gut microbiota can convert resveratrol into more bioactive metabolites such as dihydroquercetin and piceatannol. These metabolites exhibit superior bioavailability compared to the parent compound, entering the bloodstream to exert systemic anti-inflammatory and antioxidant effects [87]. A study also revealed abnormal accumulation of fatty acids in the intestines of atherosclerotic mice. In animal experiments, administering 10 mg/kg resveratrol orally twice daily for 24 weeks to ApoE^−^/^−^ mice fed a high-fat diet eliminated this accumulation. In cellular experiments, RAW264.7 macrophages treated with 65 μg/mL oleic acid for 24 h exhibited induced lipid accumulation. Co-treatment with 1.5 μg/mL resveratrol reduced lipid accumulation by promoting cholesterol reverse transport through activation of the PPARα/γ pathway [88]. Via the gut–liver axis, signals and metabolites generated by resveratrol in the intestine reach the liver through the portal vein, where they directly regulate lipid metabolism: downregulating hepatic transcription factors Hepatocyte Nuclear Factor 4 alpha (HNF-4α) and FoxO1 to reduce ApoC-III production. This accelerates clearance of triglyceride-rich lipoproteins and improves hyperlipidemia [89]; resveratrol activates the SIRT1/AMPK pathway in the liver while inhibiting inflammatory signaling pathways like NF-κB. It promotes the production of the vasodilator nitric oxide, thereby improving vascular endothelial function and reducing vascular inflammation and oxidative stress. These actions collectively exert hepatoprotective and anti- atherosclerotic effects [87]. It is noteworthy that there exists significant individual variation in gut microbiota composition, leading to differing rates of production of resveratrol metabolites such as lunularin and resulting in distinct “metabolic phenotypes.” This makes it challenging to observe consistent health effects at the population level. This will be one of the fundamental challenges encountered when translating laboratory discoveries into clinical applications [90].

Quercetin typically exists in foods as a glycoside, which is poorly absorbed directly. Ingested quercetin glycosides (such as rutin) can be deglycosylated and cleaved by gut microbiota into readily absorbed aglycones and small-molecule acids, which exhibit anti-inflammatory activity. Concurrently, quercetin and its metabolites modulate microbiota composition, reduce harmful metabolite TMAO production, and strengthen the intestinal barrier, thereby systemically alleviating chronic inflammation associated with atherosclerosis. This mechanism is supported by multi-level evidence from in vitro fermentation models, animal studies (e.g., intervention in atherosclerotic mouse models at 12.5–50 mg/kg/day), and human dietary research (e.g., consumption of quercetin-rich onions) [91]. Metabolites of quercetin absorbed through the gut reach the liver via the portal vein, where they initiate a series of regulatory actions: upregulating hepatic receptors such as LXRα and PPARγ to promote cholesterol reverse transport and enhance cholesterol efflux [92]; activate antioxidant pathways such as Nrf2; and inhibit classical inflammatory signaling pathways like HMGB1/TLR4/NF-κB, thereby reducing hepatic and systemic inflammation [93]. By improving hepatic lipid processing capacity and suppressing systemic inflammation through the gut–liver axis, quercetin reduces atherosclerosis-promoting factors at their source. However, it should be noted that most of the current supporting evidence for quercetin’s effects on atherosclerosis via the gut–liver axis comes from cellular and animal studies. Human clinical research on atherosclerosis remains limited in both number and scale, and conclusions remain inconclusive [94].

### 5.2. Carotenoids

Torularhodin is a carotenoid derived from red yeast [95]. In a male mouse model of non-alcoholic fatty liver disease (NAFLD), an innovative study employed a colon-targeted delivery strategy using electrospun microspheres (EMs-T) to administer the carotenoid torularhodin (20 mg/kg/day for 10 weeks). This approach specifically promoted the growth and enrichment of *Akkermansia* muciniphila in the gut. The enriched probiotic synthesizes increased levels of adenosylcobalamin, one of the active forms of vitamin B12 in the body, which plays a crucial role in multiple cellular metabolic reactions. Research confirms that adenosylcobalamin effectively inhibits the HIF-2α signaling pathway in intestinal epithelial cells, downregulating expression of its downstream target neuraminidase 3 (Neu3). This not only helps improve insulin resistance and metabolic disorders [96] but also indirectly alleviates pathological processes such as ceramide-promoted endothelial dysfunction, inflammatory responses, and atherosclerotic plaque instability [97]. Therefore, the mechanism by which torularhodin reduces systemic Cer levels through regulation of the gut–liver axis may exert beneficial inhibitory effects on atherosclerosis.

Crocin, the active component in saffron, also belongs to the carotenoid family and may influence the progression of atherosclerosis through the gut–liver axis. A 2022 study employed LDLR^−^/^−^ mice to establish a high-fat, high-cholesterol diet-induced atherosclerosis model, administering daily oral doses of 20 mg/kg (low dose) or 40 mg/kg (high dose) crocin for 8 weeks. Results demonstrated that crocin not only significantly reduced aortic root plaque area, collagen fiber degeneration, and CD68^+^ foam cell infiltration, but also exerted anti-atherosclerotic effects via the gut–liver axis: First, crocin enhanced intestinal-barrier function by upregulating tight junction proteins (ZO-1, occludin, claudin-3) and modulating gut microbiota composition. It increased beneficial bacteria like Lactobacillus while decreasing the Firmicutes/Bacteroides ratio. On the other hand, these intestinal improvements further systemically alleviate hepatic oxidative stress, activate the Nrf2/Keap1 antioxidant pathway, and inhibit NLRP3 inflammasome and TLR4/MyD88 signaling pathways, thereby reducing systemic inflammation levels [98].

### 5.3. Panax Ginsenosides

Panax ginsenosides (PNS) are one of the primary active components of *Panax notoginseng* [99]. While their oral absorption rate into the bloodstream is limited, they can interact extensively with the gut microbiota in the intestinal tract. In both high-fat diet (HFD)-induced and genetically obese (ob/ob) mouse models of NAFLD, oral administration of PNS (800 mg/kg/day for 8 weeks) significantly upregulated the expression of intestinal tight junction proteins (such as Claudin-1 and ZO-1), repairing the intestinal barrier damaged by factors like high-fat diets. This reduces the leakage of LPS from the gut into the liver and bloodstream [100]. Upon reaching the liver, PNS activates the AMPK signaling pathway while suppressing the pro-inflammatory NF-κB pathway. Combined with its role in reducing LPS entry into the bloodstream via the intestinal barrier, these effects significantly inhibit hepatic inflammatory responses, thereby reducing inflammatory factors entering systemic circulation at their source [101]. In ApoE^−^/^−^ mouse experiments, within atherosclerotic plaques, PNS (30–120 mg/kg/d) has been demonstrated to act as an inhibitor of hypoxia-inducible factor-1α (Hif-1α), regulating energy metabolism and polarization phenotypes of plaque macrophages while improving sphingolipid metabolism. This directly inhibits plaque progression and enhances plaque stability [102]. Furthermore, PNS optimizes gut microbiota composition by suppressing harmful bacteria and promoting beneficial bacterial growth. This approach reduces harmful metabolite production while altering the bile acid metabolic profile through microbiota-mediated BSH activity. These altered bile acids, acting as crucial signaling molecules, activate nuclear receptors in the intestine and liver (FXR and TGR5). This enables remote regulation of hepatic lipid synthesis, fatty acid oxidation, and cholesterol homeostasis, while enhancing bile acid excretion. Ultimately, this leads to lipid-lowering effects and anti-atherosclerotic benefits [103].

### 5.4. Phytoestrogens

When people consume foods like legumes, the phytoestrogen glycosides they contain enter the gut. In vitro culture experiments conducted by the team led by Lucía Vázquez revealed that phytoestrogens (Genistein) at a concentration of 32 μg/mL selectively promote the growth of beneficial bacteria such as *Lactobacillus rhamnosus* and *Faecalibacterium prausnitzii*, while inhibiting *Bacteroides fragilis* [104]. This improves gut microbiome balance, strengthens the intestinal barrier, and enhances anti-inflammatory and immune regulatory functions [105]. The intestinal bacterium *Rikenella microfusus* secretes β-galactosidase, which “clips” the poorly absorbed isoflavone glycosides, converting them into the highly active and more readily absorbed Biochanin-A (Bio-A). After intestinal absorption, Bio-A reaches the liver via the portal vein. Within hepatocytes, Bio-A directly binds to and stabilizes two key metabolic enzymes: PC and PCCA. These enzymes are essential for the tricarboxylic acid (TCA) cycle. Consequently, Bio-A promotes TCA cycle activity, leading to increased glutamate synthesis—a crucial precursor for glutathione (GSH) production. Glutathione ranks among the body’s most potent antioxidants. Elevated glutathione levels effectively mitigate oxidative stress damage to the liver, thereby protecting hepatocytes. Notably, *Rikenella microfusus* is a component of the human gut microbiota, though typically present at low abundance. However, this study found that men cohabiting with female partners exhibited significantly higher abundance of *Rikenella microfusus* in their feces compared to the control group of men living alone [106]. Indeed, oxidative stress damage in the liver is a significant risk factor promoting the onset and progression of atherosclerosis: ROS can inhibit hepatic secretion of VLDL, promoting hepatic fat accumulation and subsequently triggering lipid metabolism disorders, leading to elevated levels of low-density lipoproteins (LDLs) in the blood. Concurrently, through lipid peroxidation, it increases ox-LDL levels, promotes foam cell formation, and indirectly drives AS progression [107]. Furthermore, hepatic oxidative stress promotes atherosclerotic through mechanisms including impairment of vascular endothelial function [108], triggering systemic inflammatory responses [109], and exacerbating insulin resistance [110]. Additionally, a 2001 randomized controlled trial demonstrated that daily supplementation with soy protein containing 118 mg of isoflavones for three months significantly improved blood pressure and arterial elasticity in healthy middle-aged and elderly individuals: systolic blood pressure decreased from 130 ± 2 mmHg to 123 ± 2 mmHg, diastolic blood pressure decreased from 76 ± 1 mmHg to 72 ± 1 mmHg, and femoral-brachial pulse wave velocity improved from 11.1 ± 0.2 to 10.3 ± 0.2. However, the study also indicated that a decline in brachial artery flow-mediated vasodilation occurred only in male subjects, suggesting a potential gender-specific response [111]. Furthermore, a 2005 randomized controlled trial in postmenopausal women found that cardiovascular benefits after 12 months of supplementation with soy protein containing 99 mg of isoflavones were entirely dependent on the individual gut microbiota’s ability to convert isoflavones into equol. Blood pressure reduction and improved endothelial function were observed only in phytoestrogen producers; conversely, non-producers experienced elevated blood pressure and worsened endothelial function. These findings underscore the critical role of gut metabolic phenotypes in mediating the cardiovascular effects of isoflavones [112].

In summary, phytoestrogens (Bio-A) may exert anti-AS effects by inhibiting hepatic oxidative stress. This occurs via activation and regulation of the intestinal barrier through gut microbiota, followed by transmission to the liver via the gut–liver axis to modulate hepatic antioxidant defense.

### 5.5. Phytosterols

Phytosterols may regulate atherosclerosis progression through multiple pathways via the gut–liver axis. Core mechanisms include: within the intestine, phytosterols competitively inhibit cholesterol, reducing its micellization and absorption; simultaneously, they upregulate tight junction proteins (ZO-1, Occludin), thereby enhancing intestinal barrier function and mitigating systemic chronic inflammation triggered by intestinal toxin translocation. In a study using an ApoE^−^/^−^ mouse atherosclerosis model, we found through a 21-week dietary intervention that replacing the fat portion of a Western diet (WD) with an equivalent caloric amount of high-polyphenol rapeseed oil (HPRO) rich in β-sitosterol (4983 ppm), campesterol (3475 ppm), and stigmasterol (660 ppm) (total approx. 10,176 ppm) significantly reduced serum total cholesterol (TC) and low-density lipoprotein cholesterol (LDL-C) levels (TC decreased from 25.78 mg/dL to 17.20 mg/dL) while high-density lipoprotein cholesterol (HDL-C) increased. More importantly, the overall plaque area percentage in the aorta decreased significantly from 27.28% in the WD group to 6.00%, and the aortic diameter increased significantly at multiple sites, suggesting a reduction in arterial stenosis [113]. In vitro colonic fermentation studies indicate that phytosterols, like prebiotics, promote the growth of beneficial bacteria such as *Lactobacillus* and *Bifidobacterium*, enhance short-chain fatty acid synthesis, and exert anti-inflammatory and immunomodulatory effects [114]. Furthermore, long-term epidemiological evidence supports potential cardiovascular benefits from these mechanisms: an analysis synthesizing three prospective observational studies (encompassing 213,992 individuals) found that higher dietary intake of specific plant sterols was associated with a modest reduction in coronary artery disease risk. When comparing the highest to lowest quintiles of intake, the pooled risk ratios were: sitosterol 0.89 (0.82, 0.96), stigmasterol 0.95 (0.88, 1.02), and β-sitosterol 0.92 (0.85, 1.00). This suggests that habitual intake of these phytosterols via dietary (oral) routes may confer protective effects during long-term follow-up [115].

### 5.6. Other Phytochemicals

Berberine is an isoquinoline alkaloid extracted from plants such as *Coptis chinensis* and *Phellodendron amurense*. It reduces the conversion of phenylalanine to hippuric acid (HA) by inhibiting bacteria like *Vibrio vulnificus* [116]; it also decreases the transformation of substances like choline into TMAO by optimizing the gut microbiota [117]. Concurrently, berberine strengthens tight junctions between intestinal epithelial cells, repairing the gut barrier damaged by high-fat diets. It inhibits LPS entry into the portal venous circulation, thereby alleviating hepatic inflammatory burden [118]. Additionally, its regulation of bile acid metabolism and promotion of SCFA production constitute crucial links in influencing systemic metabolism through the enterohepatic circulation [119]. Collectively, these mechanisms converge to reduce the two core drivers of atherosclerosis: abnormal blood lipid levels and chronic systemic inflammation. In a 12-week high-fat diet study in apoE^−^/^−^ mice, twice-weekly intragastric administration of berberine (50 mg/kg) significantly reduced total cholesterol levels and decreased expression of key inflammatory cytokines (e.g., TNF-α, IL-6, MCP-1, ICAM-1) and monocyte chemotactic protein. The studies directly demonstrate that under this dosing regimen, berberine reduces arterial plaque area while increasing collagen content within plaques, thereby enhancing plaque stability [117].

In a study on high-fat diet-induced obese mice, Bellidifolin—the primary ketone component from the edible plant Mongolian Hepatitis Tea (*Swertia diluta*)—demonstrated potential to regulate lipid metabolism via the gut–liver axis. Using a male C57BL/6J mouse model, researchers first induced obesity by feeding a high-fat diet for 8 weeks, followed by a 4-week intervention with oral administration of Bellidifolin at a dose of 20 mg/kg/day. The study revealed that this compound improved gut microbiota composition by reducing the abundance of Firmicutes and related pro-inflammatory families (such as *Lactobacillaceae* and *Clostridiaceae*), while promoting bile acid metabolism. Specifically, it increased the excretion of secondary bile acids and hydrophilic non-12-OH bile acids (e.g., β-MCA, Tβ-MCA, TUDCA, etc.) in feces, ultimately reducing body weight, fat accumulation, hepatic steatosis, and serum lipid levels. Furthermore, network pharmacology analysis suggested that ketone components in *Swertia diluta* are associated with lipid metabolism and atherosclerotic pathological processes. Although this study did not directly validate effects on atherosclerosis, given that bellidifolin significantly improves lipid profiles, reduces hepatic steatosis, and modulates gut–liver axis function—all critical components in atherosclerosis progression—this compound may indirectly exert anti-atherosclerotic effects by regulating the gut–liver axis and improving systemic metabolic disorders. However, its specific effects and mechanisms require further experimental validation [120].

## 6. Discussion

Atherosclerosis, as a complex chronic metabolic inflammatory disease, involves multiple pathways in its pathogenesis, including lipid metabolism disorders, immune inflammatory responses, and gut microbiota imbalance. In recent years, the gut–liver axis—a critical bridge connecting gut microbiota, hepatic metabolism, and systemic inflammation—has garnered increasing attention for its role in the development of atherosclerosis. This review systematically examines the potential mechanisms by which phytochemicals intervene in atherosclerosis through modulation of the gut–liver axis, revealing their cardiovascular protective potential at multiple targets and levels.

Although extensive preclinical research has revealed potential mechanisms by which phytochemicals intervene in atherosclerosis through the gut–liver axis, offering new perspectives for nutritional interventions in cardiovascular disease, translating these findings into reliable human clinical applications still faces a series of challenges. First, a significant gap exists between animal models and human applications. Current mechanism studies primarily rely on mouse models, whose gut microbiota, immune systems, and metabolic pathways exhibit fundamental differences from humans. The human gut microbiome displays extremely high individual heterogeneity, influenced by genetics, diet, medications, and the environment, directly leading to substantial variations in intervention outcomes. Second, most active compounds (e.g., resveratrol, quercetin aglycone) suffer from low oral bioavailability and complex intestinal metabolism. Achieving effects observed in animal studies often requires doses far exceeding dietary levels, raising concerns about long-term safety. Although novel technologies like nano delivery and structural modification hold promise for enhancing targeting, their costs, manufacturing complexity, and regulatory pathways remain practical barriers. Furthermore, potential adverse reactions and long-term safety cannot be overlooked. High doses or prolonged use of phytochemical concentrates may carry risks, including potential endocrine disruption from phytoestrogens, mineral absorption interference from polyphenols, and unpredictable active or toxic metabolites produced by gut microbiota metabolism. Additionally, interactions with conventional drugs (e.g., affecting hepatic enzyme activity) must be evaluated for clinical application. Finally, plant chemicals lack unified standards for sourcing, extraction processes, and formulation types. Their in vivo activity is highly dependent on metabolic conversion by gut microbiota, with the same precursor yielding multiple metabolites exhibiting distinct activities. Current evaluation systems based solely on raw material content fail to accurately reflect actual biological efficacy, making it difficult to compare and integrate findings from different studies.

## 7. Conclusions

Human life and health depend on dietary nutrients. Our research demonstrates a close interaction between dietary phytochemicals and atherosclerosis. These bioactive compounds primarily influence atherosclerosis progression through the gut–liver axis by modulating gut microbiota composition, repairing and strengthening the intestinal barrier, regulating bile acid metabolism, and exerting anti-inflammatory and antioxidant effects. Simultaneously, phytochemicals synergistically combat atherosclerosis through multiple pathways by promoting beneficial bacterial proliferation, inhibiting pathogenic bacteria growth, and increasing levels of beneficial metabolites such as short-chain fatty acids. Therefore, incorporating foods rich in phytochemicals into daily diets as a strategy for home-based prevention and dietary intervention against atherosclerosis warrants further research and application.

## Data Availability

No new data were created or analyzed in this study. Data sharing is not applicable to this article.

## Supplementary Materials

The following supporting information can be downloaded at: https://www.mdpi.com/article/10.3390/nu18020188/s1, Table S1: Checklist of ltems to Include When Reporting a Systematic Review Involving a Network Meta-analysis. Table S2: Changes in Gut Bacterial Abundance in Atherosclerosis and Their Regulation by Phytochemicals. Table S3: Dietary Sources, Content, and Biological Activities of Selected Plant Compounds. Table S4: Summary of Mechanisms by Which Phytochemicals Intervene in Atherosclerosis Through the Gut-Liver Axis.

## Abbreviations

The following abbreviations are used in this manuscript:

AS	Atherosclerosis
TMAO	Trimethylamine *N*-oxide
SCFAs	Short-chain fatty acids
LPS	Lipopolysaccharide
FXR	Farnesoid X receptor
TGR5	Takeda G protein-coupled receptor 5
PPARs	Peroxisome proliferator-activated receptors
IL	Interleukin (e.g., IL-1β, IL-6)
TNF-α	Tumor necrosis factor-alpha
NF-κB	Nuclear factor kappa-light-chain-enhancer of activated B cells
NLRP3	NLR family pyrin domain containing 3
AMPK	AMP-activated protein kinase
Nrf2	Nuclear factor erythroid 2–related factor 2
ApoE	Apolipoprotein E
LDLR	Low-density lipoprotein receptor
HDL-C	High-density lipoprotein cholesterol
LDL-C	Low-density lipoprotein cholesterol
TC	Total cholesterol
VLDL	Very low-density lipoprotein
ox-LDL	Oxidized low-density lipoprotein
CYP7A1	Cholesterol 7-alpha hydroxylase
BSH	Bile salt hydrolase
ABCA1/ABCG1	ATP-binding cassette transporter A1/G1
LXRα	Liver X receptor alpha
HMG-CoA	3-hydroxy-3-methylglutaryl-coenzyme A
MMP	Matrix metalloproteinase
CD	Cluster of differentiation
sIgA	Secretory immunoglobulin A
AMPs	Antimicrobial peptides
HDAC	Histone deacetylase
GPR	G protein-coupled receptor
TLR4	Toll-like receptor 4
MCP-1	Monocyte chemoattractant protein-1
ICAM-1	Intercellular adhesion molecule-1
HFD	High-fat diet
NAFLD	Non-alcoholic fatty liver disease
RCT	Reverse cholesterol transport
GSH	Glutathione
ROS	Reactive oxygen species
MDA	Malondialdehyde
SOD	Superoxide dismutase
CAT	Catalase
GPX	Glutathione peroxidase
