# Supplementary material for: Effects of Phytochemicals on Atherosclerosis: Based on the Gut–Liver Axis
Source: Nutrients. 2026 Jan 6;18(2):188. doi: 10.3390/nu18020188 (PMC12844917; doi:10.3390/nu18020188)
Supplement: Supplementary file 1 [file nutrients-18-00188-s001.zip › nutrients-4055779-supplementary.pdf]

Supplementary Information Contents

Table S1. Checklist of ltems to Include When Reporting a Systematic Review Involving a Network Meta-analysis.

Screening Dimension	Inclusion Criteria	Exclusion Criteria
Study Type	1. Animal studies in vivo (especially atherosclerosis models, e.g., $ApoE^{-/-}$ or $LDLR^{-/-}$ mice).<br>2. Human clinical studies (randomized controlled trials, cohort studies, etc.).<br>3. In vitro cell studies elucidating gut-liver axis mechanisms (e.g., co-culture models).	1. Purely computational (in silico) simulation studies.<br>2. Conference abstracts only, unpublished dissertations.<br>3. Case reports irrelevant to the topic.
Subject of Study	1. Studies focusing on atherosclerosis, hyperlipidemia, or related cardiovascular endpoints.<br>2. Studies involving gut microbiota or liver metabolism.	Disease models irrelevant to the topic (e.g., studies on obesity or diabetes without linkage to atherosclerosis).
Intervention	1. Natural phytochemicals from dietary sources or herbal medicine (e.g., polyphenols, alkaloids, flavonoids) or their extracts.<br>2. Interventions aimed at influencing liver metabolism or systemic inflammation via modulating gut microbiota.	1. Single, purified synthetic compounds or chemical drugs.<br>2. Mixed formulations of phytochemicals with synthetic chemicals (e.g., sugars, pharmaceuticals).<br>3. Compounds with unspecified origins.
Mechanism of Action	1. Studies explicitly involving the gut-liver axis, i.e., investigating how phytochemicals affect atherosclerosis by altering gut microbiota composition/function, thereby influencing hepatic lipid metabolism, bile acid cycling, inflammation, etc.<br>2. Mechanisms involving the regulation of nuclear receptors (e.g., PPARs, LXRs) or inflammatory pathways like PCSK9.	Studies describing only the direct lipid-lowering or anti-inflammatory effects of phytochemicals without exploring indirect mechanisms mediated by gut microbiota.
Outcome Measures	1. Contains key atherosclerotic endpoints: aortic plaque area, blood lipid profiles (TC, TG, LDL-C, HDL-C), systemic inflammatory markers (e.g., TNF-α, IL-6).<br>2. Contains gut microbiota analysis (e.g., 16S rRNA sequencing) and/or measurement of relevant microbial metabolites (e.g., SCFAs, secondary bile acids).	Only general physiological parameters (e.g., body weight) measured, lacking the core outcome indicators mentioned above.
Study Design	Original research articles (providing complete methodology and data).	Reviews, systematic reviews, meta-analyses, book chapters, commentaries.
Publication Characteristics	Peer-reviewed published literature.	Literature for which the full text is inaccessible.

Table S2. Changes in Gut Bacterial Abundance in Atherosclerosis and Their Regulation by Phytochemicals

Bacterial Type	Genus/Species	Change in Relative Abundance in Atherosclerosis	Examples of Modulating Phytochemicals
Anti-inflammatory Bacteria	Akkermansia muciniphila	Decreased	Green tea polyphenols (EGCG), Licorice flavonoids, Resveratrol, Ginsenosides, Quercetin
Anti-inflammatory Bacteria	Bifidobacterium spp.	Decreased	Phytosterols, certain prebiotic-like phytochemicals
Anti-inflammatory Bacteria	Faecalibacterium spp.	Decreased	Phytoestrogens (e.g., Genistein)
Anti-inflammatory Bacteria	Lactobacillus spp.	Decreased	Crocin, Phytoestrogens, Phytosterols
Pro-inflammatory Bacteria	Proteobacteria	Increased	Not explicitly specified
Pro-inflammatory Bacteria	Streptococcus spp.	Increased	Not explicitly specified
Pro-inflammatory Bacteria	Bacteroides fragilis	Increased	Phytoestrogens (Genistein) can inhibit its growth
Pro-inflammatory Bacteria	Clostridiaceae	Increased (associated with pro-inflammation)	Bellidifolin can reduce its abundance
Other Relevant Bacteria	Rikenella microfusus	Increased in specific populations (e.g., men cohabiting with women)	Involved in phytoestrogen metabolism, promoting conversion to active forms

Table S3. Dietary Sources, Content, and Biological Activities of Selected Plant Compounds

**Plant Compound**	**Common Dietary Sources & Content**	**Main Bioactivities (Based on Current Research)**
Protocatechuic Acid	1. Star Anise: 32.20 mg<br>2. Chicory (Green): 21.79 mg<br>3. Black Olives: 6.00 mg<br>4. Onion (Red): 2.00 mg	Exhibits antioxidant and anti-inflammatory effects, protects vascular endothelial function. In vitro studies show antibacterial activity.
Resveratrol	1. Grape skin: 50-100 mg / 100 g<br>2. Blueberries: 0.4 mg / 100 g<br>3. Cranberries: 1.9 mg / 100 g<br>4. Peanuts: 0.15 mg / 100 g	Possesses antioxidant and anti-inflammatory properties. Research suggests potential protective effects in cell and animal models against cardiovascular diseases, neurodegenerative diseases, diabetes, obesity, and various cancers.
Quercetin	1. Onion: 8.59 mg<br>2. Broccoli: 8.09 mg<br>3. Lotus Root: 7.55 mg	A potent antioxidant. Some epidemiological studies indicate an inverse association between its intake and coronary heart disease risk.
Torularhodin	red yeast dry biomass: 25.9 mg/100g.	Antioxidant, anticancer, and antibacterial effects.
Crocin	1. Saffron (Stigma): 10%<br>2. Gardenia (Fruit Pulp): 0.5%-1.2%	Studies indicate potential therapeutic effects for central nervous system diseases and cardiovascular diseases. Also exhibits broad pharmacological activities including anticancer, anti-inflammatory, antioxidant, hepatoprotective, and antidiabetic effects.
Panax Ginsenosides	1. 6-year-old ginseng root: 59.77 mg/g<br>2. 5-year-old ginseng flower: 113.78 mg/g<br>3. 5-year-old ginseng fibrous root: 75.01 mg/g	Research shows antioxidant and anti-inflammatory properties.
Phytoestrogen (Genistein)	1. Dried Soy Milk Cream: Mean 37.53 μg/g<br>2. Soybean: Mean 35.54 μg/g<br>3. Soybean Sprouts: Mean 31.26 μg/g	Estrogen and endocrine regulation, cardiovascular system protection, metabolic and inflammatory regulation
Berberine	Coptis chinensis: 5.90% to 9.32%	Lower blood sugar, regulate blood lipids, and exhibit antibacterial activity
Bellidifolin	Swertia diluta: 32.50 mg/g	Regulates lipid metabolism, improves gut microbiota, modulates bile acid metabolism, and protects liver function.

Table S4. Summary of Mechanisms by Which Phytochemicals Intervene in Atherosclerosis Through the Gut-Liver Axis

Phytochemical	Class	Mechanisms via the Gut-Liver Axis (Summary)	Primary Evidence
Protocatechuic Acid (PCA)	Polyphenol	Enhance intestinal barrier function and reduce inflammatory factors; activate the NRF2 pathway to alleviate hepatic oxidative stress and lipotoxicity; inhibit cholesterol synthesis and absorption.	Animal studies (piglets, mice)
Resveratrol	Polyphenol	Promotes the growth of beneficial bacteria (Akkermansia) and their metabolism into bioactive compounds; enhances cholesterol reverse transport via PPARα/γ; activates SIRT1/AMPK, inhibits NF-κB, and improves vascular function.	Animal and cell studies
Quercetin	Polyphenol	Metabolized by gut microbiota into active aglycones; reduces TMAO and enhances intestinal barrier function; activates LXRα/PPARγ to promote cholesterol efflux; inhibits the HMGB1/TLR4/NF-κB inflammatory pathway.	Animal studies, in vitro fermentation models
Torularhodin	Carotenoid	Targeted delivery promotes Akkermansia proliferation, increases adenosylcobalamin synthesis, inhibits the HIF-2α/Neu3 pathway, and improves insulin resistance and endothelial function.	Animal study (NAFLD model)
Crocin	Carotenoid	Increase tight junction proteins to optimize microbial community structure; activate the Nrf2/Keap1 antioxidant pathway while inhibiting NLRP3 and TLR4/MyD88 inflammatory pathways.	Animal study ($LDLR^{-/-}$ mice)
Panax notoginseng Saponins (PNS)	Saponins	Repair the intestinal barrier to reduce LPS translocation; activate AMPK to inhibit NF-κB; regulate microbiota and bile acid metabolism by activating FXR/TGR5 to modulate hepatic lipid metabolism.	Animal studies (multiple models)
Genistein	Phytoestrogens	Promotes the growth of beneficial bacteria; metabolized by the microbiota into highly active Biochanin-A, enhancing the liver's antioxidant capacity (boosting GSH) and inhibiting oxidative stress-driven AS.	In vitro culture & animal studies
β-Sitosterol	Phytosterols	Competitively inhibits cholesterol absorption; Strengthens the intestinal barrier; Promotes probiotic growth and short-chain fatty acid (SCFA) production; Long-term consumption is associated with reduced risk of coronary heart disease.	Animal studies & cohort studies
Berberine	Alkaloid	Inhibit the metabolism of TMAO by harmful bacteria; Repair the intestinal barrier; Regulate bile acid metabolism; Lower blood lipids and inflammatory factors, enhancing plaque stability.	Animal study ($ApoE^{-/-}$ mice)
Bellidifolin	Other (Ketone)	Regulates gut microbiota composition and promotes bile acid excretion; improves blood lipids and hepatic steatosis, potentially exerting anti-AS effects indirectly through enhanced metabolism.	Animal study (obesity model)
